# The chemical geographies of misoprostol: Spatializing abortion access from the biochemical to the global

**DOI:** 10.1080/24694452.2023.2242453

**Published:** 2023-09-27

**Authors:** Cordelia Freeman, Sandra Rodríguez

**Affiliations:** Department of Geography, University of Exeter. Amory Building, Rennes Drive, Exeter, EX4 4RJ; Pontificia Universidad Católica del Perú

**Keywords:** Abortion, Chemical Geography, Latin America, Pharmaceuticals, Reproductive Justice

## Abstract

*C*_22_*W*_38_*O*_5_ is a chemical that travels. Better known as misoprostol, it was designed as a stomach ulcer drug but is now used around the world as an abortion pill due to the self-experimentation of Latin American communities who were seeking ways to end unwanted pregnancies. We develop a chemical geography approach to misoprostol that allows us to scale inward to understand the chemical properties of this medication while also being able to scale out to understand how medicinal effects are interwoven with and determined by global politics. Misoprostol as a chemical alone does not guarantee a successful abortion and instead ‘scaffolding’ in the form of mobility and information is required to transform misoprostol from a chemical to a safe and effective technology of abortion. First, we examine how misoprostol is moved by feminist networks in Mexico and Peru. Second, we argue that in order to be useful it is not enough just to access the pills, information on how to use them is required. These themes culminate in our contribution of ‘pharmacokinetical geographies’; the micro-geography of the placement of pharmaceuticals in and on a body and its ramifications. The chemical geographies of misoprostol tell a story of power, bodily autonomy, and resistance.

## Introduction

Chemical geography is a relatively new but loosely formed body of scholarship that examines the relationships between chemicals and the wider world. The geography literature to date tends to focus on industrial chemicals and pollutants and the effects they go on to have as they move through environments, but pharmaceuticals have received far less attention. In this paper we use the abortion pill misoprostol as a vehicle to argue for a chemical geography that can help us to analyse the story of a chemical that begins in a lab, travels across continents and through and into bodies in experimental ways, is facilitated by a range of actors, and has spatial engagements with bodily interiors.

Abortion is a highly common procedure in Latin America; every year 6.5 million abortions take place across the continent (Bearak et al. 2020). But what is an incredibly safe procedure when performed correctly can become a dangerous one when facilities and information are not available, and so nearly one million women are hospitalized every year due to complications resulting from an unsafe abortion (Zamberlin and Raiher 2009). However, the way in which these abortions are conducted has transformed in recent decades due to the introduction of new technologies, new forms of communication, and the mobilization of feminist groups and activists (Burton 2017a).

The most influential new technology is misoprostol, a medication designed for stomach ulcers that is now used to terminate pregnancies globally in medical spaces and informal settings. It is this ‘double life’ (De Zordo 2016) that makes misoprostol use possible but that also conditions the (im)possibilities of its circulation. The development of misoprostol as an abortion pill tells a specific gendered and racialized story. Across Latin America and the world, many chemicals, plants, and techniques have been used by women and pregnant people who want to end their pregnancies (Schiebinger 2004; Ross and Solinger 2017; Solinger 1994). Misoprostol is not the only chemical important to reproductive lives nor is it the only technique of abortion, but it has emerged from a multiplicity of techniques to become the dominant method of self-managed and clinical abortions due to being safe, effective, and mobile. The increased use of medications to induce abortions has made self-managed abortions safer than ever before (Raymond et al. 2019, Grossman et al. 2018), but significant legal, economic, geographic, and informational barriers to safely accessing misoprostol remain. The existence of the pill alone is not a guarantee of an effective abortion, instead those seeking a safe abortion require knowledge of how to access it, how to use the correct dosage, what to expect, what to do in case of an emergency, and how to avoid prosecution. It is this information and support, predominantly led by formal and informal feminist networks, that transforms misoprostol from a chemical to a safe technology of abortion.

The basis for this paper is a multi-sited research project in Mexico and Peru, two countries that can be considered ‘states of uncare’ regarding abortion (Duffy, Freeman and Rodríguez 2023). Abortion is largely criminalized across Latin America and where exceptions do allow for an abortion, it remains very difficult to access in practice (Zamberlin and Raiher 2009). In Mexico, abortion is severely restricted in the majority of states but has been decriminalized in Mexico City since 2007, with some other states following suit since 2019. It is also legal in the case of rape at a federal level, but some states have hardened their anti-abortion stance in response to pushes for liberalisation, and even where people do meet the strict criteria for a legal abortion, stigma and the lack of infrastructure make access highly challenging (Singer 2018; 2019). In September 2021, the Supreme Court passed a series of historic rulings to guarantee access to abortions as part of women’s constitutional right to reproductive freedom and autonomy. While significant, it is too early to see the full effects of these legislative changes.

In Peru, abortion is technically legal where there is a threat to the life or health of the woman. However, these circumstances are exceptional and Peru has one of the most restrictive abortion frameworks in Latin America (Motta and Salazar 2019). Despite being highly restricted, Peru has an abortion rate higher than many countries where abortion is legal (Bernabé Ortiz et al. 2009), with dire health consequences that manifest in high rates of hospitalizations and maternal deaths from unsafe procedures (Taype and Merino 2016). Since 1997 it has been a criminal act for health professionals to not report a suspected abortion to the authorities, making the emergency room of public hospitals the departure point for the legal prosecution of women (Rousseau 2007). In this context, activists and abortion providers have stepped in to provide safe, effective, and empathetic abortion care where that has been denied by the state (Duffy, Freeman and Rodriguez 2022; Belfrage 2022). In this paper we particularly focus on the use of misoprostol by *acompañantes:* people who provide accompaniment, guidance, and support -whether virtual or physical-throughout the abortion process.

This paper uses three themes to analyse the transformation of misoprostol from a chemical to a safe technology for abortion. We begin by explaining the biography of misoprostol and show the margins of legality regarding the ‘double life’ of misoprostol and its idiosyncrasy as a legal/illegal product that became an abortion pill after Latin American women experimented with it on their own bodies. We then examine two forms of multiscalar ‘scaffolding’ to understand abortion access in restricted settings. The term ‘scaffold’ in a scientific sense refers to molecular structure but in this paper we seek to extend scaffolding out beyond the chemical structure of misoprostol itself to how the chemical is manufactured, sold, bought, circulates between and within bodies, ingested, and how their effects are understood. The scaffolding metaphor reflects our multi-scalar perspective to understand the chemical geographies of misoprostol from the scaffolding of its molecular structure to its global politics. This multi-scalar perspective draws on feminist geography literature on scale that rejects a flat ontology or nested notion of scale in favour of a ‘double helix’ intertwining of scale (Pain and Smith 2008). The first type of scaffolding is mobility whereby we trace the material journey of pills from manufacture to consumption, an often sticky and low-tech journey that requires complex negotiation between actors to source the pills and keep them in circulation. The second type of scaffolding is information, and we explore how feminist networks generate, translate, and disseminate knowledge of how to use misoprostol.

These themes braid together and culminate in our theoretical contribution of ‘pharmacokinetical geographies’. The scientific knowledge formed through Latin American women’s experimentation and the legal context they have abortions in, combined with the movement of misoprostol and the dissemination of information of how to administer it, has disrupted the original or intended micro-geography of its movement and resulted in a very specific dosage of misoprostol. Pharmacokinetics is the study of how drugs are absorbed, distributed, metabolized, and excreted (Wagner 1981) and we argue that this forms part of the ‘micro-geography’ of misoprostol in terms of how and where it enters the body. This is a way to ‘scale in’ (Agard-Jones 2013) to biochemical effects but can never be separated from broader socio-political relations that modulate knowledge valuation, regulatory and legal frameworks, and economic conditions. Examining misoprostol in this way, as a chemical element that through scaffolding becomes a technology of abortion, reframes abortion as a structural, collective endeavour, rather than a hyper-neoliberal, individualised ‘choice’ (Tajer 2019).

## Methods

This research is based on 29 interviews with people involved in abortion access and activism in Mexico and Peru. These participants ranged from employees of multinational NGOs with outward-facing roles who were involved in lobbying, working with governments, or healthcare providers to ‘on-the-ground’ activists fighting for and realising abortion provision in their local communities, including *acompañantes*. Participants were recruited through purposive sampling (Crookes and Davies 1998), in order to specifically select participants who had the necessary knowledge and were contacted through our existing research networks and through contact details found through internet searching.

The interviews were conducted between January 2020 and March 2021 with five in-person and the remaining 24 through virtual teleconferencing software when the covid pandemic began. The interviews were open-ended discussions, and while the two of us had an agreed interview guide, we used this loosely so that the interviews were more like a conversation and could be directed by what the participants had to say (Roulston and Choi 2018). The interview questions were about abortion access broadly and particularly focused on the circulations and mobilities of information and misoprostol pills that allow those seeking abortions to receive safe healthcare at home or with a medical provider.

The interviews were transcribed and coded by both authors using a loose coding frame that was firmed up using inductive coding so that we were led by the data (Linneberg and Korsgaard 2019). All interviews took place in Spanish and any quotes used here were translated into English by the authors. Ethical approval was granted through The University of Exeter’s ethical review board on the Department of Geography and confidentiality was of utmost importance with all data stored securely and full anonymity ensured.

### The chemical geographies of pharmaceuticals

Chemical geographies is a small and relatively new area of research with disagreements about its current status. Even within one jointly authored paper different authors alternately argue that it is “not a new subfield nor does it seek to become one” and “a nascent geographical subfield” (Romero et al. 2017, 159, 162). What the authors do agree on is that chemical geographies aims to understand the connections between chemicals and the world in a broad sense. A core contribution of this literature has been an emphasis on relations. Andrew Barry (2017) has argued that social scientists, including geographers, have neglected chemistry and he has sought to trace chemical histories to show why this neglect matters. For instance, Barry explains the term ‘chemical space’ which has been used by pharmaceutical chemists to refer to out-reaching legal and economic relations of chemicals, not just their internal spaces. This builds on his work on ‘informed materials’ where he argues that “molecules should not be viewed as discrete objects, but as constituted in their relations to complex informational and material environments” (Barry 2005, 52). Barry’s work has been useful at showing how molecules exist beyond the laboratory and are ‘informed’ by a range of regulatory, legal, and economic factors.

Chemical geography literature to date has centered around industrial chemicals, pollution, and waste, but little work has included the chemical geographies of pharmaceuticals. We make a case for bringing pharmaceuticals into chemical geography by drawing on medical anthropology scholarship on the ‘social lives of medicines’. In their agenda-setting book, Whyte, Van der Geest and Hardon (2002) call for a focus on the *materiality* of medicines as they move between settings and actors. In the scholarship preceding and following this text, work on the social lives of medicines has explored the ‘thinginess’ of traditional medicines and laboratory-created pharmaceuticals, the details of those who create, manufacture, and market medical materials, and the experiences of those administering and receiving medicines. However, as Whyte, Van der Geest and Hardon (2002, 3) state, they “are more concerned with their social uses and consequences, than with their chemical structure and biological effects.” Our argument here is that the social uses and consequences of medicines cannot be understood without full consideration of their chemical structure and biological effects. Chemical geography and STS (Science and Technology Studies) literatures, with their spatio-temporal sensibilities, aid us in making this argument.

STS scholarship, particularly feminist and postcolonial STS, has explored how science and knowledge are value-laden and spatially contingent (Tsing 2005; Harding 2009; 2014). Feminist and postcolonial STS, while having their internal differences, “decenter and parochialize dominant ways of thinking about the production of scientific and technological knowledge and their familiar philosophic assumptions” (Harding 2009, 418). STS challenges a linear ‘progress narrative’ and disrupts ideas of authority in scientific, medical, and health knowledges (Knopes 2019). These bodies of literature are informative to our chemical geography analysis of the pharmaceutical product misoprostol because, as soon as it left the lab, it has been Latin American women’s bodies that have been its site of experimentation, and racist, colonial, patriarchal assumptions about knowledge and science have affected how misoprostol has been understood and hence used. Chemicals are highly uneven in their spatial concentrations (Patchin 2020), but so too is knowledge and understanding of them.

The pulling together of chemical geography, medical anthropology, and STS literatures informs our chemical geography of misoprostol. This lens allows us to explore the histories and effects of pharmaceuticals from their chemical structures right through environments to the legacies they go on to have. By taking this one chemical, and tracing its spatio-temporal biography and its relations with people, bodies, vehicles, digital spaces, and other forms of infrastructures, we show the ‘scaffolding’ involved in transforming misoprostol from a chemical designed to treat stomach ulcers to an abortion pill. This multi-scalar journey culminates in the pharmacokinetic geography of misoprostol as we explore how the pill travels from the laboratory into the user’s bloodstream. We begin with an explanation of how misoprostol came to be an abortion medication.

### The biography of misoprostol

The chemical *C*_22_*H*_38_*O*_5_, better known as misoprostol, was developed in 1973 by Searle (now Pfizer) for the treatment of gastric ulcers, receiving United States Food & Drug Administration approval in 1988 (Löwy and Dias Villela Corrêa 2020; MacDonald 2021). Marketed with the brand name cytotec, misoprostol is a synthetic analogue of prostaglandin E1 (Barbosa and Arilha 1993) and has effects beyond the treatment of gastrointestinal problems. It has prostaglandin-related side effects, particularly vomiting, diarrhoea, fever and chills (Billings 2004), but it also causes abortions. This is due to its chemical effects of softening the cervix and provoking uterine contractions. Misoprostol has therefore been termed a ‘pharmaceutical outlaw’ (MacDonald 2021) that has a ‘double life’ (De Zordo 2016), as it became used as an abortifacient (ie an agent that induces abortion). But how did this ‘double life’ originate?

This story begins in Brazil in the mid 1980s where misoprostol was being sold for stomach ulcers. The packaging of misoprostol comes with a clear warning – ‘not be used by pregnant women’, with an image of a heavily pregnant cartoon woman with a red line across – and it was this warning that served as an advertisement to those seeking to end their pregnancy (Nations et al. 1997). This warning offered a solution to Brazilian women amongst other abortion techniques including other medications, herbs, and physical methods. While it is unclear exactly when or where misoprostol began being used as an abortifacient, by the late 1980s and into the early 1990s its use was widespread in Brazil; the ‘cradle’ of misoprostol’s transformation into an abortion pill (De Zordo 2016).

In contrast to countless other experiments when Latin American communities have been experimented on by scientists primarily from the global North, Brazilians were ‘auto-experimenting’ and autonomously testing these pills for their own ends. Auto-experimentation here does not indicate a purely individual practice however, as it was based on shared and collective information and experience. The knowledge these women developed in the ‘southern periphery’ fed back into global North medical knowledge, where ‘traditional’ scientific research took place which led to misoprostol being used as an abortifacient all over the world as a ‘legitimate’ medication (De Zordo 2016; Hardon and Sanabria 2017). While the global South can be a space for pharmaceutical experimentation, knowledge was ‘remade’ in northern scientific spaces to become legitimised in a global North-centric culture that only accepts Western (or ‘Dominant’) science as ‘real’ (Harding 2014; Liboiron 2021). Latin American women were therefore ‘rewriting the script’ of misoprostol (MacDonald 2021), and their bodies were, and continue to be, their own laboratories for working out how to best administer the medication for a safe abortion as well as a site of ongoing re-articulation for the politics of knowledge around abortifacients.

Through this auto-experimentation and subsequent formal scientific testing, an effective regimen was developed. As one interviewee in Peru explained, “it has taken years, and unfortunately many difficult situations for many women, to find the ideal route, and the right dose”. In the early 1990s, research papers did not even see misoprostol as an abortifacient on its own. It was described as having “some uterine effects” but “not effective in inducing complete abortion” (Costa and Vessey 1993, 1260) and as a “a weak abortifacient, but the induced bleeding justifies women to ask for medical assistance and they then can obtain access to curettage at public hospitals” (Coelho et al. 1994, 102). It was therefore not seen as a technique for a complete abortion but as “a “passport” for obtaining abortions at public health services” (Barbosa and Arilha 1993, 239).

The current situation is radically different as misoprostol went from being ineffective, to an effective and safe process if administered correctly (Kapp and Lohr 2020). Misoprostol alone has efficacy rates of 88-93 percent in clinical studies with the reported incidence of serious complications at just 0.2 percent (Cohen et al. 2005; Raymond, Harrison, and Weaver 2019). This makes misoprostol a safe and reasonably effective method of abortion where mifepristone is not accessible (Rowlands 2012).^1^ It was only as greater knowledge and more accurate information about how to access and use misoprostol was shared between those seeking abortions that it could be used as a standalone technique for a complete abortion. This spread of information cannot be attributed to a single source, instead it was pharmacies, doctors, the manufacturer, women themselves, and the media who all shared information about the drug with each other and beyond (Barbosa and Arilha 1993; Fernández Vázquez and Szwarc 2018). This meant that by the late 1990s, misoprostol was one of the most commonly used abortifacients in Latin America (Drovetta 2015). The development of the World Health Organization (WHO) protocol on safe use then provided a legitimized source that facilitated clear and accurate guidance of how to use misoprostol for abortion seekers, activists, and providers.

As the transformation of misoprostol through mediation with Latin American bodies shows, ‘toxicity’ is contextual and shifting. Toxicity has been seen as central to reproductive justice (Liboiron, Tironi and Calvillo 2018), but this conversation has generally focused on whether or not people are able to reproduce or the facilitation of safe reproduction (Patchin 2020; Mansfield 2012), not on the potentials for toxicity to *bring about* reproductive justice. Misoprostol is labeled with the warning of ‘reproductive toxicity’ but for someone seeking an abortion, ‘toxicity’ can mean freedom and a solution to an unwanted pregnancy. Chemicals are not inherently ‘good’ or ‘bad’, instead they are ambivalent in their ability to be at once enabling and harmful. Misoprostol overflows the boundaries of its intended use: it complicates the notion of ‘harm’, and poses challenges to its regulation. A chemical that is a ‘bad actor’ (Liboiron 2016) for some, is a liberatory one for others. Through domesticating the ‘toxicity’ of misoprostol - on their own bodies - women and pregnant people harnessed the toxic side-effects to become intended effects.

Misoprostol now has multiple potential uses beyond treating NSAID related ulcers. Since 2005, misoprostol and mifepristone have been classed as essential medicines by the the World Health Organization (WHO) and in 2009 this was amended so that misoprostol was included on the list specifically for the treatment of incomplete abortions (Zamberlin, Romero, and Ramos 2012). On the basis of the scientific consensus of its efficacy to provoke safe abortions, its use has been expanded for other gynaecological purposes, and it is now used by medical providers to manage miscarriages, prevent postpartum haemorrhage, and make labour easier by softening the cervix (Elati and Weeks 2009; Stephenson and Wing 2015). Searle’s patent of cytotec ending in 2000 and then the adding of misoprostol to the WHO essential medications list led to misoprostol being registered for use in more countries around the world and the availability of generic versions (Atukunda et al. 2015). These redevelopments in the biography of misoprostol illustrate how chemicals and experimentation with them are not fixed, rather they are re-evaluated for biological, political, and cultural reasons.

Misoprostol as a chemical therefore inhabits an unusual space. Its marginality as legal for stomach ulcers but in many countries illegal for abortions means that it is available. In reconstructing the genealogy of misoprostol as an abortion pill, the link between its double life and the politics of knowledge around its use comes to the fore. Where the state fails to provide abortion healthcare and actively criminalizes abortion care, the knowledge around misoprostol as an abortion pill was created at the margins, and this was possible due to the complex legal status of the medication. This same complex legal status has, however, reinforced stigma and ignorance of misoprostol. This differentiates it from the other abortion medication, mifepristone, which, as its only purpose is abortions, is much more challenging to find in places where abortion is not legal. Because misoprostol is registered for other medical uses, “that makes it easier for the drug to exist in pharmacies, it is restricted, but at least it does exist [in Mexico],” explained one interviewee. Misoprostol is also marginal in the sense that it blurs the borders of abortion as its effects are indistinguishable from a miscarriage, which is a significant benefit in restricted settings where abortions are punished through custodial sentences (Freeman 2017). In the rest of this paper we expand on the double life of misoprostol to trace the chemical geographies of the medication and argue that *C*_22_*H*_38_*O*_5_ has become a safe and effective technology of abortion through two elements of scaffolding: mobility and information. To begin, how does misoprostol move in contexts that criminalize its use for abortions?

### Mobility: Moving misoprostol in restricted environments

Misoprostol has undoubtedly transformed abortion access across Latin America. But, in restricted settings, the existence of the chemical in pill form does not mean it is always easy to access (Freeman 2020). Geographers have begun to examine how the mobility of abortion pills has fundamentally transformed the spatiality of abortion access. Rather than people moving from locations where they cannot access an abortion to one where they can (Freeman 2017), the movement of pills to people can support them to end their pregnancies on their own terms (Calkin 2020; Calkin and Freeman 2019; Calkin, Freeman and Moore 2022; Engle 2022; Freeman 2020). Mobility is central to understanding abortion they argue, because, “across scales, from the clinic to the nation-state, bodies, pills and knowledge are on the move in ways that reflect, reinforce and contest power relations” (Calkin, Freeman and Moore 2022, 1416). The transformation of abortion access, then, has not been simple.

To be useful, misoprostol needs to undertake a multi-scalar journey to leave the factories in which it is manufactured and travel all the way into the bloodstream of the person seeking to end their pregnancy. There are a range of barriers that impede this from happening, such as not knowing misoprostol exists or where to find it, physically being unable to travel to access pills or have them travel, pharmacies refusing to sell pills without a prescription, and the difficulties of finding pills that are genuine, affordable, and within-date. But the materiality of misoprostol facilitates the movement of the pills: they are small, thermo-stable, have a long shelf life at room temperature when stored in their aluminium blister packets, and are easily transported across jurisdictions (Elati and Weeks 2009; Calkin 2020). But they do not move by themselves, instead actors and infrastructure are required, and it is these that provide the scaffolding to create the chemical mobilities of misoprostol. We focus here on feminist networks (a spectrum of actors from multinational NGOs to small, community-based groups of volunteers) and the strategies they use to move misoprostol in Mexico and Peru. Feminist networks work to prevent ‘coagulation’, a term used by Sodero (2019) to refer to disruptions of circulation, at two key moments of misoprostol’s movement: sourcing the pills and facilitating their mobility.

Many interviewees we spoke to received donations of misoprostol from multinational NGOs and global social marketing organizations (Belfrage forthcoming). But, for those without these donations, there are three primary sources of misoprostol in Mexico and Peru: pharmacies, the black market, and laboratories. In many parts of Latin America, misoprostol can be purchased from pharmacies and while pharmacy staff are usually required to ask for a prescription signed by a doctor, in practice they are often sold without one (Lara et al. 2006). According to one interviewee in Peru, pharmacies are risky “because without a prescription they won’t sell them to you or if they do, it’s at a very expensive price, or you’re gambling with the risk that they’re adulterated or out of date”. But there are pharmacies that sell misoprostol, without requiring a prescription, and at a relatively fair price. Feminist networks therefore formulate strategies to find out which pharmacies these are. One Peruvian feminist group described how they created a ‘heat map’ so they knew where such pharmacies are, whether a prescription is required, and how much they sell the pills for. One *acompañante* in Peru explained how she regularly went to the same pharmacy, where “they always sold me them [misoprostol pills], they just looked at me weird (laughs) but they sold me them”. Another explained how “we actually look for places. Basically, we knock on doors. We verify that it is a safe place. We make sure it’s open all the time to sell us the medication; that yes, they will sell them to us. Usually they charge us more.” Our research found that these raised prices are not uncommon with pharmacies charging two to five times higher with no prescription compared to with a prescription. Pharmacies, despite their complications, are therefore an important and valuable source that allows for the mobility of misoprostol.

Pharmacies are not the only source of misoprostol. There is a thriving and lucrative black market for the pills advertised through flyers, stickers, and graffiti telling you who to call, websites that tout their wares, herbal markets that will provide them if asked, and sellers who meet buyers in public spaces, particularly around university campuses (Gynuity Health Projects 2007). On the black market prices can be much higher (Zamberlin, Romero, and Ramos 2012), pills may come within ‘packages’ (often marketed as combi-packs) that contain other unnecessary medicines, they may never arrive, or may be adulterated or ineffective (Drovetta 2015). For instance, some of these ‘pills’ are in fact sugar or flour or medications that provoke vaginal bleeding but are not abortifacients (Gynuity Health Projects 2007). Black market websites are becoming increasingly savvy with their design, as one interviewee in Peru described, making their sites look like feminist abortion sites and copying the language and style of prominent pro-abortion campaigns. Receiving ineffective pills or never receiving them at all is particularly an issue with something as time-sensitive as abortion. Moreover, these black-market sellers, whether in-person or online, too often provide incorrect information about how to administer the pills. The role of feminist networks is to intervene in this problematic sourcing by warning people of the risks of black market sellers and providing information on how to access misoprostol through other routes.

A third source cuts out pharmacies and the black market as some networks buy directly from laboratories. Particularly in Peru, the ‘*paraíso del miso*’ [paradise of misoprostol], the increasing manufacture of misoprostol in Latin America has led to the shortening of these circuits. Local pharmaceutical companies make generic misoprostol in multiple Latin American countries including Brazil, Colombia, and Peru (Gynuity Health Projects 2007). This is not to say that these are necessarily feminist spaces of emancipation, and *acompañantes* in Mexico are concerned about the power and profits gained by pharmaceutical companies (Belfrage forthcoming). These companies are producing legal misoprostol, due to its ‘legitimacy’ for treating stomach ulcers, but those seeking to buy them as abortifacients can take advantage of this hyper-local provenance. While individuals themselves can source misoprostol directly from pharmacies or black-market sellers, activists play a unique role in building relationships with laboratories to source the pills. In both Mexico and Peru, abortion activists and providers act as regular customers and so develop contacts who can sell them the pills directly or support them in buying large numbers cheaply. This means there are scalar geographies to the mobility of misoprostol. Sometimes their mobile journey is long: originating in a factory in India where they are bought by an international NGO who sends them to a national NGO within Latin America who then distributes them to activist groups who can pass them onto people seeking an abortion. Along this journey the pills may coagulate and become stuck in a certain location. One *acompañante* in Mexico, for example, was struggling to find misoprostol but her contacts in other Mexican states had received large donations and could therefore disperse them into areas of need. But, sometimes the journey is short as they are produced and sold directly to an activist who gives them to the abortion seeker. The simplification of this journey reduces the delays or obstacles that can lead to coagulation.

Abortion activists develop strategies to move the pills as efficiently as possible given the socio-legal context. Through interviews we learnt about techniques based on low-tech infrastructures required to move the pills in situations where they are not legal. For example, one *acompañante* in Peru told us of one case during the COVID-19 pandemic when a woman was quarantined inside her house with her husband and so could not leave to buy misoprostol. The *acompañante* managed to buy the pills and smuggle them into the woman’s house under the guise of grocery shopping.

The woman was then able to take the pills and tell her husband, who was not the father, that she was having a miscarriage. One *acompañante* in Mexico described ‘camouflaging’ pills by hiding them within a gift or teddy bear so that they could be sent to someone without their parents or partner knowing. Other interviewees in Mexico described asking a brother to take the pills to someone’s house, sending them to a friend’s house, leaving them at a hotel reception desk, or using a post office box where someone can collect their pills without having them sent directly to their home. Moving the pills to rural areas can be particularly challenging and activists have to find creative solutions. As one interviewee in Peru explained, “one thing that I have only done once but others in the group have done more often, is to send the pills [to rural areas] by bus, to give them to the driver who doesn’t know what he’s carrying”. However the pills arrive, the *acompañante* is then able to support the person receiving them through the abortion by telephone.

Feminist networks across and beyond Latin America have emerged as a crucial part of the landscape in facilitating the movement of the pills from a range of sources, even by developing direct relationships with laboratories, to those who are seeking abortions. This is a complex web of mobility and any flows are far from smooth. In places where misoprostol cannot be legally used for abortions the pills are forced to coagulate, they disappear, they are adulterated, and low-tech tools such as smuggling pills in groceries and on buses are needed to push the pills through these messy mobile circuits. The strategies outlined here for making misoprostol move have been central to increasing its availability. However, accessing the pill is not enough if you don’t know how to take it and crucially, where and how to place it in the body, and so accurate information is required to be able to administer the medication effectively. As the following section argues, the dissemination of information is the necessary second part of the scaffolding that transforms misoprostol from a chemical to a safe and effective technology of abortion.

### Information: Translating and disseminating the regimen

Information about how to safely and effectively use misoprostol to end a pregnancy is too often flawed and potentially dangerous. While there is a safe and effective regimen, knowledge of it is not available to all. A diverse range of actors in Mexico and Peru therefore utilise strategies to generate, translate, and disseminate the information about how to safely induce a complete abortion using misoprostol amongst a wider group of people seeking abortions. Before we explain these strategies, we first set out the barriers to accessing information on how to use misoprostol.

Due to Searle/Pfizer not applying for approval for misoprostol to be used as an abortion medication, it is primarily used off-label (Gemzell-Danielsson, Fiala, and Weeks 2007). Off-label drug use is a very common and normalised practice but it does lead to challenges for the effective use of misoprostol. The 200-microgram tablet size it is manufactured in is not appropriate for many uses and the information that comes with the pills will not be accurate for an abortion, particularly when the dosage depends on the week of gestation (Elati and Weeks 2009). Those reading the accompanying guidance for the treatment of stomach ulcers will not learn that misoprostol is ineffective in terminating a very early pregnancy, nor that it can be dangerous at term. Pharmacy staff who sell misoprostol can only officially access recommended dosages for treating stomach ulcers. For example, in Mexico, the *Diccionario de Especialidades Farmacéuticas*, includes no obstetric information about misoprostol and few pharmacy workers are trained on the multiple uses of the medication (Billings et al. 2009). In their study of pharmacy staff, Lara et al. (2006, 2011) found that just 15-17 percent of staff recommended a dosage that was potentially effective for an abortion. Staff were sharing inaccurate information and were recommending hormonal injections, which are not an abortifacient, more often than they were recommending misoprostol. Being able to buy misoprostol from a pharmacy does not therefore guarantee a safe and effective abortion. People seeking an abortion require accurate and detailed information about how to administer the pills and this is where the role of feminist networks has been crucial.

There are three roles that feminist networks take in sharing information about how to use misoprostol: generation, translation, and dissemination. First, as we have shown, the knowledge of how to use misoprostol as an abortifacient originated in Brazil. This information initially circulated in informal ways, and was partial and patchy. Over time, those administering the medication, on themselves or others, gradually learnt which strategies of dosage were most effective and this information fed from their bodies to scientific understandings of the drug. The exact dose of misoprostol recommended for an abortion is not static, and activists and providers have continued to develop the regimen. An *acompañante* in Mexico explained that part of her generation of knowledge was unlearning the ‘vices’ she had learnt from purely medical approaches to abortion that only consider physical aspects of the process, and ignore emotional experiences and socio-legal contexts.

Many groups follow the WHO recommendation which is 12 pills taken across three doses, but others use their experiences of accompaniment to adapt this. For example, one group in Mexico recommends two doses of 4 pills while another uses just one dose of four pills. Others have found that certain plants such as rue or Andean mint aid the process and so their protocol includes them. One interviewee in Mexico who uses plants explained how some of this knowledge came from ancestral knowledge, some from books about herbs in Mexico, some from academic research, and then trying things out in a way that she described as “it wasn’t so much testing as it was like ah, look, this works...It has been a bit intuitive but also from looking for information that tells me ah, this is [...] not such a good option.” Another group recommends against eating chocolate while going through the procedure because in their experience it can reduce the efficacy of misoprostol while other groups say this has not been their experience. A key finding has been that misoprostol is not effective in the first six weeks of gestation and this has fed into many groups’ protocols.

There is a type of tacit knowledge that is built up through accompaniment work to recognise when bleeding is too heavy or when the gestational sac has passed. The experience of accompanying abortions is an embodied one, that Zurbriggen (2019) terms ‘*actos corpo-aborteros*’ (bodily-abortive acts) and she stresses the importance of emotions and corporality in the construction of knowledges. An interviewee in Peru described the kind of knowledge that they gain from doing the accompaniment work as ‘*casuística*’; a type of knowledge learnt from one case and applied to others. The framing of *casuístic* knowledge is important because it takes seriously the type of knowledge or ‘lay expertise’ generated and shared by activists that don’t have formal medical training (McReynolds-Pérez 2017). By showing the wealth of knowledge generated through tacit, embodied, or *casuístic* means, we can disrupt the “familiar philosophic assumptions” of Dominant science (Harding 2009, 418).

Abortion knowledge generation is not just used to help those having an abortion at that moment but moves through feminist networks. Interviews showed that there is international sharing of information about how to effectively administer misoprostol based on collating experiences of how it has been used across Latin America. There are groups in key locations such as Ecuador, Mexico, Argentina, Chile, and Peru who have shared their expertise and experiences with one another. One interviewee in Mexico described this sharing of knowledge as incredibly ‘generous’ as everyone involved is committed to improving abortion access for all. These links have been created and strengthened through in-person events where activists travel from across the continent, and occasionally outside Latin America. As Burton (2017b, 103) argues, a feature of *acompañante* groups is their “incessant reflection on their own practice”. This sharing of experience and learning from and reflecting on one’s own experiences has been important for developing the most effective regimen but also what the risks are and when someone should go to the hospital due to excessive bleeding. In practice then, misoprostol’s toxicity was not neatly domesticated just through developing an effective regimen; the dosage is constantly redeveloped and knowledge of it needs to be continuously disseminated.

Second, once this *casuístic* knowledge has been generated, complex medical information needs to be translated in a way that makes it accessible for women and pregnant people to understand what to do and what to expect during an abortion with misoprostol. One interviewee explicitly described her role as an *acompañante* as a translator of complex medical information, saying “what do you do with the information if you don’t know how to apply it, or how to use it so that you abort safely? If you don’t know how to use misoprostol, or where, or with whom, or anything”. Part of this translation is developing a verbal and visual language that does not feel detached. Another *acompañante* in Peru explained that her organization “wanted to create resources that spoke about abortion... without being cold or distant” and they do this through their individual skills. They have an obstetrician who understands the medical side, a journalist who ‘translates’ that to a broader audience, and creative members who turn that information into audiovisual materials.

Third, this translated guidance then needs to be disseminated so that it can reach and be used by those using misoprostol for abortions. A range of low-tech strategies including stickers in the streets and bathroom stalls, social media groups and websites, handbooks, and hotlines have all formed part of information dissemination. Handbooks are physical or online guides or manuals that clearly explain how to safely have an abortion with misoprostol, and have often been created by abortion hotlines (Drovetta 2015). In our interviews, the importance of the Peruvian hotline manual was mentioned by multiple interviewees. *Hablemos de aborto y misoprostol* [Let’s talk about abortion and misoprostol] was first published in 2014 and provides the protocol for using misoprostol through text and illustrations (Drovetta 2015). As of 2023, this manual has been downloaded over 73,000 times and some interviewees even had it to hand when we spoke to them. These manuals explain in detail how to safely use misoprostol in a way that is accessible to those who might be completely unfamiliar with the process, including those who may struggle with the language. For *acompañantes* we spoke to, it was often the entry point to understanding the safe and effective dose and regimen of misoprostol for an abortion. One interviewee believed that the information about how to abort with misoprostol has always circulated but the Peruvian manual made it much clearer and more accessible. These fairly low-tech and highly shareable handbooks have changed the landscape of information about misoprostol across Latin America, particularly in urban areas, by translating complex medical information into accessible guides for self-managing an abortion.

Hotlines are telephone services where those seeking information about abortion can call up and speak to someone about their situation, about their legal rights, and about how to procure and self-manage an abortion. The telephone operators are trained in providing evidence-based information in clear and simple ways and give advice on whether the caller may need to seek medical attention (Dzuba, Winikoff, and Peña 2013). Like the handbooks they tend to exist on national scales across Latin America, but those who receive the calls often hear from people in other countries. Also like the handbooks, they are low-tech and this has been key to their success. For example, in 2010 the Ecuador hotline was shut down by a court order but it was immediately able to start up again with a new number (Drovetta 2015). These hotlines provide an important service as ‘conduits’ (McReynolds-Pérez 2017), in that they are able to disseminate information that is personalised, personable, and up-to-date.

As a result of these information sharing strategies, those seeking abortions in Mexico and Peru have greater awareness of their rights and options. Until recently, women in Latin America have typically had poor knowledge of abortion medications before becoming pregnant (Zamberlin, Romero, and Ramos 2012). But interviews showed that generally information in the public consciousness is increasing and more abortion seekers are calling saying they want ‘misoprostol’. One interviewee in Peru explained that “I feel like now the information is more visible. Because two years ago they would say to me ‘they told me about some pills but I don’t know what they’re called’, or they simply didn’t know anything: whereas now women are coming better prepared with information, they may have already had an ultrasound and located the [misoprostol] pills”. Interviewees also spoke about the transformative power of being supported through an abortion with feminist accompaniment. One *acompañante* in Mexico found that after the abortion women wanted to become involved in activism, help to share pills, and financially support others.

Information is therefore a crucial part of the scaffolding process. Without it, someone seeking an abortion may have the pills in their hand but not the knowledge of how to take them in a way that will result in a successful abortion. With abortion restricted across most of Mexico and Peru, actors have mobilised to generate, translate, and disseminate information about how to use misoprostol to those seeking an abortion. To do this they have adopted low-tech strategies including handbooks and hotlines to provide visually clear, evidence-based, and personalised information. This is an ongoing process and as new knowledge is generated or as socio-legal contexts change, so too do translation and dissemination strategies. So far, we have considered the movement of misoprostol and knowledge surrounding it at global, national, and local scales. However, to fully understand the chemical geographies of misoprostol we need to scale inwards further to the body, mouth, and blood.

### Pharmacokinetical geographies: Scaling into the bloodstream

Brazilian women’s bodies were a key site of experimentation to develop the most effective regimen for aborting with misoprostol, but this process of experimentation did not end in the 1990s. This knowledge was not discovered in the laboratories of the global North but through the auto-experimentation of women on their own bodies on the one hand and through *casuística* or close observation by providers and activists on the other. In the medical anthropology literature ‘wrong use’ of medicine is seen as a problematic issue that can make medicines ineffective or dangerous (Geest, Whyte and Hardon 1996). In the case of misoprostol, it was ‘wrong use’ that transformed lay and medical understandings of ‘toxic’ misoprostol and created a new technology of abortion. Through auto-experimentation, users and providers have designed an effective regimen with misoprostol and this regimen requires its own relationship with the user’s body.

There is a chemical geography to how misoprostol affects the body at a biochemical level. Where and how the chemical enters the body affects the effectiveness of the chemical as a technology of abortion. The half-life of an 800μg oral dose of misoprostol is 1.0401±0.5090h, for a sublingual dose (placed under the tongue) it is 0.8542±0.1170h, and for a buccal dose (places between the gum and cheek) it is 0.8365±0.1346h.^2^ In practical terms this means that an oral dose has an eight minute onset of action and a duration of two hours, a sublingual dose is eleven minutes onset and three hours duration, and so on, which signifies that to be most effective, misoprostol should be left to dissolve under the tongue or between the cheek and gum rather than swallowed. Oral administration is less effective and is more likely to lead to side effects whereas sublingual administration leads to the shortest time to reach peak level in the user’s body (Tang and Ho 2006). Biological factors such as the abundant blood supply under the tongue and the optimal pH in the mouth likely lead to the suitability of buccal and sublingual placement (ibid). These highly specific spatial differences in the placement of misoprostol have led to what we term ‘pharmacokinetical geographies’. This contribution brings together auto-experimentation and the politics of scientific knowledge, the mobility of pharmaceuticals, and information on how to use them.

Pharmacokinetics refers to the movement of drugs into the body, also referred to in pharmacology as ADME (absorption, distribution, metabolism, and excretion). Routes of administration dictate how the body is exposed to and affected by pharmaceutical compounds. Pharmacokinetical geography therefore refers to this micro-geography of the placement of pharmaceuticals in and on a body, how it enters the bloodstream and then muscles and organs, and the ramifications of this, particularly for efficacy. The scientific knowledge of placing the pills under the tongue or between the cheek and gum came not from laboratory testing of half-lives but from lived experience and experimentation on the part of Latin American women. This information needs to be shared clearly with abortion seekers and the aforementioned handbooks provide visual clarity to ensure the effectiveness of misoprostol. Online guides and handbooks commonly include drawings of wide-open mouths with pills tucked between the cheeks or under tongues. This takes the mobility of pills and information further to understand the mobility of the chemical molecules as they are placed into the mouth, enter the bloodstream of the user, and provoke uterine contractions.

Pharmacokinetical geography is of course ‘informed’ by wider regimes. For instance, there may be more-than-biological reasons for why pharmaceuticals might be entered into certain parts of the body over others. In settings where abortion is restricted it is advised that misoprostol is used sublingually or buccally rather than vaginally, not because it is more effective that way but because remnants of misoprostol located in the vagina during a pelvic examination could lead to criminal prosecution (Jayaweera, Moseson, and Gerdts 2020). The final part of ADME, excretion, is particularly pertinent in contexts where abortion is illegal. The chemical properties of misoprostol mean that it undergoes rapid esterification to its free acid and is further metabolized in several tissues and then excreted mainly in urine. The consequence is that the short serum half-life of misoprostol means that it cannot be detected through medical testing in the plasma or urine and the pharmacokinetics of misoprostol leaving the body rapidly and stealthily is as important as how misoprostol enters the body. The porosity of bodies means they are in constant exchange with environments, including legal ones (Hardon and Sanabria 2017).

Within pharmacokinetics, the acronym ADME is sometimes expanded to account for the potential or real toxicity of the compound, becoming ADMET or ADME-Tox. As discussed earlier, toxicity is not absolute but depends on the intended consequence of a compound, and is determined by pharmacokinetics. Importantly, toxicity is not separate from ADME, and how a pharmaceutical is absorbed, distributed, metabolised, and excreted, determines whether it works or harms. Studies have determined the lethal dose of misoprostol (using LD50) in rats and mice but no lethal dose has been determined for humans. An overdose may increase the risk of adverse effects including abdominal pain, diarrhoea, and fever but through interviews, participants in both Mexico and Peru more commonly pointed to the adverse effect being an unsuccessful abortion. If too few pills can be located, not enough are taken, or if they have been tampered with or expired, then the complete abortion is less likely. Interviewees also discussed the practices of care to manage the potential toxicity of misoprostol for example recommending that people eat a light meal so that they are less likely to have diarrhoea or using herbs to ease the pain.

Moreover, as geographers have considered the temporality of toxic materials (Mansfield 2017), pharmacokinetical geographies are temporal as well as spatial. Time is an important factor that needs to be considered in chemical geographies, especially when chemicals are uneven in their spatial and temporal distributions (Murphy 2013). This can be extended to these pharmacokinetical geographies as on a much more micro-scale, the way that a chemical enters the body dictates the temporality of the chemical within the body and thereby its rate and how effective it is. The speed of ADME is informed by the administration of pharmaceuticals at micro-scales. A focus on pharmacokinetics follows Agard-Jones’ (2013, 192) interest in ‘scaling inwards’, “recognizing the multiple levels at which our material entanglements—be they cellular, chemical, or commercial—might be connected to global politics”. The chemical effects are taking place at the micro-scale but whether and how these effects take place are ‘informed’ by their relations with complex environments (Barry 2005).

This information is multi-scalar, with political pressure affecting what information is known, transnational sharing of knowledge, local bespoke protocols of dosage, and the discoveries about the pharmacokinetical effects of misoprostol on a micro-scale. However, this is not a ‘dripping down’ from the scale of the global to the biochemical; understandings of misoprostol cut across scale and the biochemical is intertwined with and feeds into the global. Agard-Jones (2013, 187) calls this a “kind of scale-jumping...moving us from the innards of embodiment to the space of global capital”. The rearticulation of misoprostol is produced through the ‘friction’ of the interaction of a range of actors, encompassing global scientific elites and the auto-experimentation of Latin American women (Tsing 2005). Drawing from this, our contribution of pharmacokinetical geographies furthers both medical anthropology and chemical geography by emphasizing how micro-level analyses are not just useful, but vital to understand chemical entanglements, the politics of knowledge, and global flows of pharmaceuticals.

## Conclusion

Our chemical geographies perspective of misoprostol has made three key contributions. Our first is empirical. As our work on misoprostol has shown, it is a technology that has transformed access to safe abortions across Latin America, but this has not happened in a vacuum. Through the ‘scaffolding’ of mobility and information, we have highlighted the people, particularly feminist activists and those who have experimented with misoprostol, who have developed strategies to transport the medication and to generate, translate, and disseminate information about how to use it effectively. Second, contributing to feminist geographical work on scale, we speak to the burgeoning area of abortion geography, where scholars have begun to explore the scalar dimensions of abortion from the body, to the clinic, to the nation-state (Calkin, Freeman and Moore 2022). We have made a case for an even more granular thinking: to the molecular and the cellular. Our contribution of pharmacokinetical geographies shows that we cannot think of these scales as discrete and bounded, rather the chemical entanglements of misoprostol are at once spatialized through biochemical processes and global politics. This takes the materiality of misoprostol seriously, as a pill that is fabricated in factories, that gets posted and passed between hands, that dissolves under tongues, and metabolizes in the body causing uterine contractions. Our contribution to feminist geography therefore encompasses scholarship on a critical feminist issue as well as feminist thinking on scale.

Our third key contribution is to the scholarship on the chemical geographies of pharmaceuticals. While chemical geographies has to date largely sidelined pharmaceuticals, we have brought in medical anthropology and STS literatures to spatialize work on pharmaceuticals and show that geographers are ideally located to provide important insights into the power, inequities, and spatio-temporal geographies of these chemical lives. We have been able to focus on the literal movement of one pharmaceutical product but we have also critically interrogated medical knowledges. We have illustrated how misoprostol knowledges were partially formed in ‘traditional’, ‘scientific’ spaces, but were radically reimagined through the auto-experimentation of people searching for ways to end their pregnancies and through *casuístic* knowledge when other routes for abortion are denied by the state. Our chemical geographies perspective of misoprostol therefore provides a framework that highlights the agency of the people involved in the development of this medication as an abortifacient, rescales the geography of abortion in feminist geography, and respatializes chemical geographies literature to consider the multi-scalar politics of pharmaceuticals.

## Data Availability

Given the nature of our data, interview transcripts are not publicly available. Please contact the authors to discuss data access further.

## Footnotes

1 The ‘gold standard’ medication abortion procedure is to use misoprostol with mifepristone, an antiprogestogen that provokes uterine contractions, as the efficacy rate is as high as 97 percent (Dzuba, Winikoff, and Peña 2013). Mifepristone is much harder to locate in contexts where abortion is illegal specifically because its primary use is as an abortifacient, unlike misoprostol which travels more easily due to its ‘double life’.

2
https://pubchem.ncbi.nlm.nih.gov/compound/Misoprostol#section=Biological-Half-Life
